# Use of 3D-Printed Patient-Specific Guide for Posterior Bone Block Procedure in Patients With Posterior Shoulder Instability: A Technical Note

**DOI:** 10.7759/cureus.95286

**Published:** 2025-10-24

**Authors:** Bernard De Geofroy, Damien Lami, Jean-Charles Grillo, Thibaut Poujade, Camille Choufani

**Affiliations:** 1 Orthopaedics and Traumatology, Laveran Military Hospital, Marseille, ATF; 2 Orthopaedic Surgery, Institut du Mouvement et de l'appareil Locomoteur, Marseille, FRA; 3 Orthopaedics and Traumatology, Pole ORTHO-SPORT Les Fleurs, Toulon, FRA; 4 Laboratory, Newclip Technics, Haute Goulaine, FRA; 5 Orthopaedics and Traumatology, Sainte-Anne Military Hospital, Toulon, FRA

**Keywords:** patient specific 3d-printed guide, patient-specific instrumentation (psi), posterior bone block, posterior shoulder instability, shoulder injuries in young athletes

## Abstract

Posterior shoulder bone block using an iliac graft is one of the standard techniques for posterior glenohumeral instability. Whether performed openly or arthroscopically, it appears to deliver the same results but must be performed with technical rigor to avoid the disadvantages (protrusion and impingement with long-term arthrosic risk). We hypothesised that patient-specific instrumentation (PSI) combined with an ancillary device could help optimise the surgical procedure. The technique presented here describes, for the first time, the installation of a posterior shoulder bone block using an iliac crest graft, with a customised cutting guide and HyLa (Latarjet Hybrid, NEWCLIP®) placement ancillary, enabling both open and arthroscopic surgery.

## Introduction

Posterior shoulder bone block using the iliac crest is an uncommon technique first described by Ilfeld in 1943 [1]. Since then, various posterior stabilization techniques have been described with comparable outcomes [2,3], the most widespread being iliac crest stabilization. Recent technological advances have made it possible to perform this procedure arthroscopically, with results similar to those obtained using open techniques [4]. The goal of these procedures is to limit posterior dislocation of the humeral head by filling the posterior glenoid defect and blocking posterior translation. However, functional outcomes remain disappointing in cases of hyperlaxity or multidirectional instability, with a high recurrence rate and the onset of symptomatic shoulder arthritis [5,6]. In traumatic instability, incorrect positioning of the bone block (overhang) may further accelerate osteoarthritic progression [7].

Three-dimensional (3D) printing has been increasingly applied in orthopaedic surgery [8]. Patient-specific instrumentation (PSI) based on preoperative imaging has shown benefits for implant positioning in hip and knee surgery [9,10], and has also been applied to shoulder arthroplasty, although not yet to posterior shoulder instability [11-13]. The use of PSI may allow more precise graft and screw positioning, reducing the risk of complications associated with conventional techniques [14]. Building on these advantages, we describe a new open surgical technique for the posterior shoulder bone block. This procedure combines a custom 3D-printed cutting guide for iliac crest harvesting with a reusable HyLA placement ancillary (Hybrid Latarjet, NEWCLIP®, Haute-Goulaine, France), aiming to improve accuracy, reproducibility, and patient outcomes.

## Technical report

Technique

Concept and Materials

To perform a Latarjet anterior shoulder bone block combined with open graft harvesting and arthroscopic placement, we used the HyLa ancillary described by Lami et al. [15]. Using this tool, we reproduced the concept for an open posterior bone block technique. To further secure the procedure, a patient-specific cutting guide was employed to allow standardized graft harvesting adapted to both the glenoid morphology and the placement of ancillary. The main pitfalls we sought to avoid with this approach, including inadequate graft size, inappropriate graft morphology, malpositioning of the bone block, and the risk of anterior impingement from excessively long screws-are summarized in Table 1.

**Table 1:** Pitfalls and theoretical advantages provided by the use of PSI-guided posterior iliac bone block technique HyLa: Latarjet Hybrid, NEWCLIP® (Haute-Goulaine, France), PSI: Patient-specific instrumentation.

Pitfalls	Advantages
Harvesting an inadequately sized graft with risk of fracture during screw fixation in case of a small graft	Standardized graft harvesting (size and morphology adapted to the glenoid)
Harvesting a graft with inadequate morphology compared to the glenoid shape	Preoperative morphometric planning and analysis of the iliac crest and glenoid
Insufficient spacing between screws leading to a risk of graft fracture	Pre-positioning of pins during graft harvesting, ensuring reproducible spacing and placement of screws on the graft
Bone block positioned too laterally (overhanging) or too medially	HyLa 2-in-1 graft holding device allows controlled positioning and prevents graft overhang with an adjustable intra-articular tab (blade)
Screws of excessive length with risk of anterior impingement	Anticipation of screw length to avoid anterior protrusion and conflict with the subscapularis muscle

**Figure 1:**
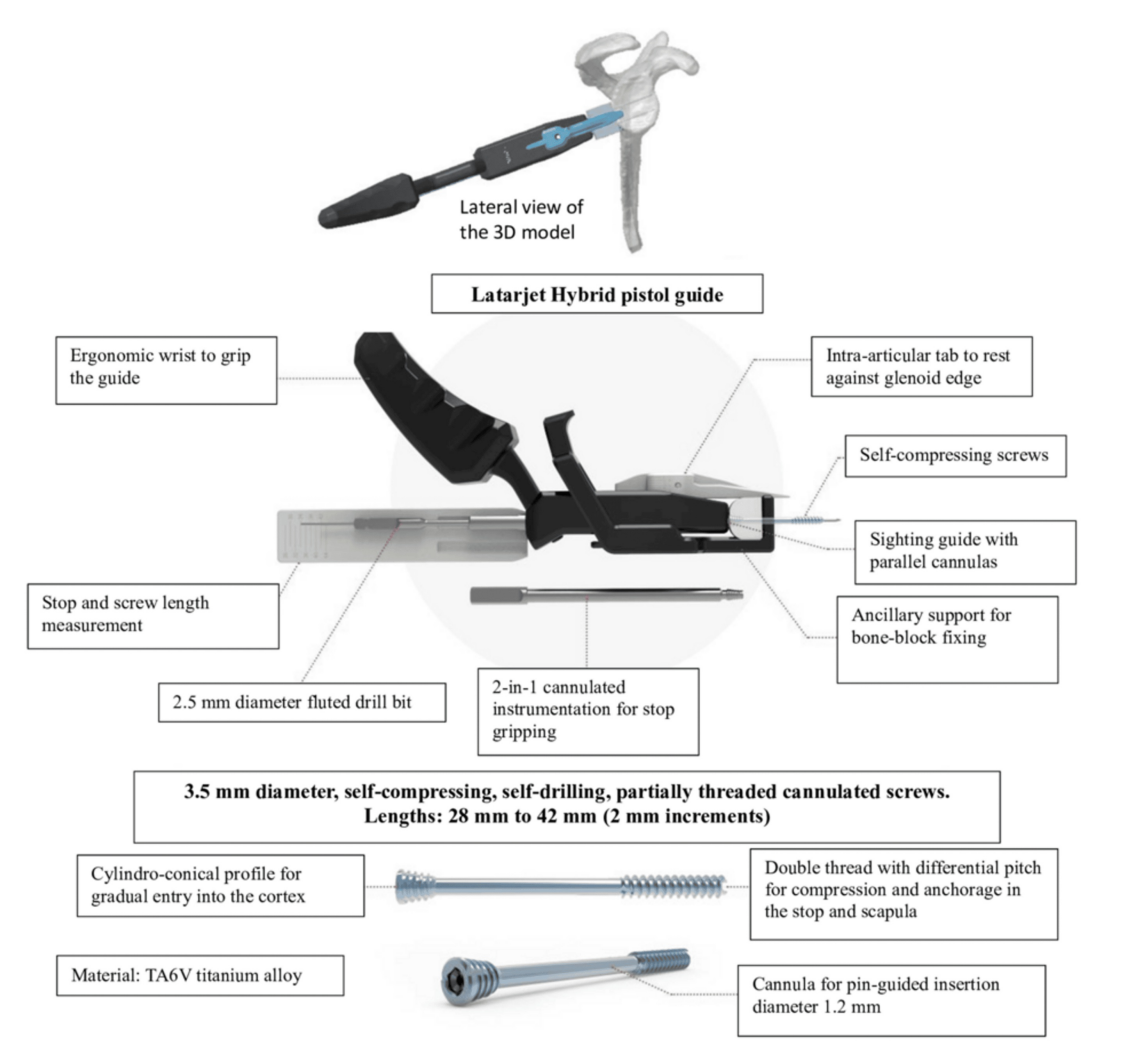
Virtual bone-block positioning using the HyLa guide: guide description and particularities Virtual bone-block positioning using the HyLa guide. The illustration shows the main features of the device: cylindro-conical profile for gradual entry into the cortex, intra-articular tab resting against the glenoid edge, ergonomic handle for optimal grip, and self-compressing cannulated screws (3.5 mm diameter, 28–42 mm length, TA6V titanium alloy) with double thread for differential compression and anchorage. Additional instrumentation includes a 2.5 mm fluted drill bit, sighting guide with parallel cannulas, and ancillary support for bone-block fixation. HyLa: Hybrid Latarjet, NEWCLIP®, Haute-Goulaine, France.

The HyLa 2-in-1 graft holding system provides precise control during graft placement and ensures correct positioning by preventing graft protrusion through its adjustable intra-articular tab (blade) (Figure 1).

The open technique will be described in this article.

Indications for Posterior Bone Block

Posterior bone block procedures were indicated in cases of recurrent posterior shoulder instability associated with a documented posterior glenoid bone loss greater than 10%, or with a failed conservative treatment after at least six months of dedicated rehabilitation. All patients presented with recurrent posterior subluxations or dislocations confirmed clinically and radiographically (CT scan). The three patients included in this technical note comprised two active-duty military personnel and one amateur rugby player, all engaged in high-demand upper limb activities. Each patient provided written informed consent for both the surgical procedure and the open-access publication of anonymized data and images.

Development of the Patient-Specific Cutting Guide

**Figure 2:**
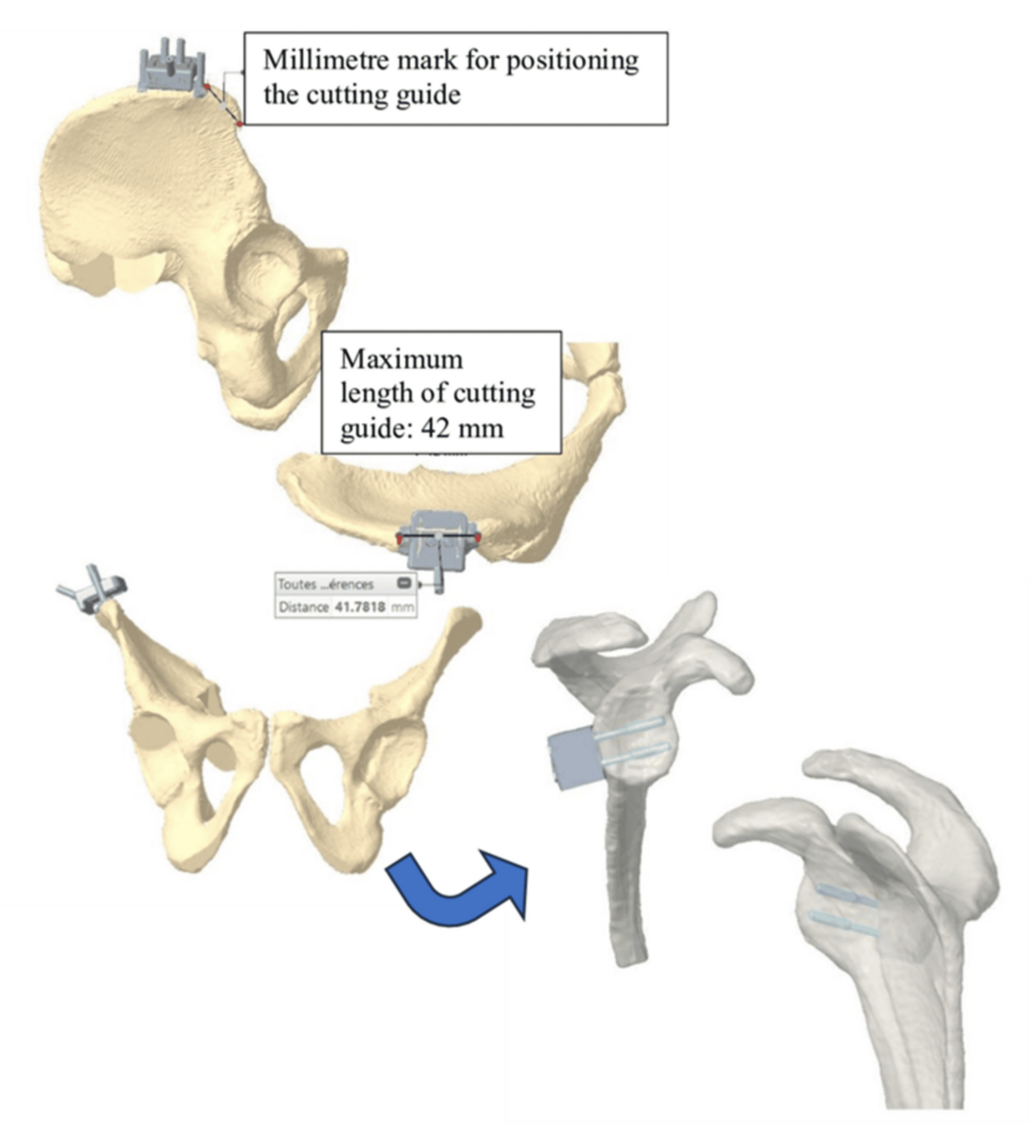
Positioning of the iliac crest graft cutting guide and 3D virtual analysis of bone-block positioning and screw length Positioning of the iliac crest graft cutting guide with millimetre landmarks for accurate placement. The 3D virtual analysis illustrates bone-block positioning on the glenoid and the preoperative determination of screw length (maximum cutting guide length: 42 mm).

Scans were acquired according to the recommendations of Newclip Technics, and the resulting DICOM (Digital Imaging and Communications in Medicine) files from the shoulder and pelvis were used to generate three-dimensional reconstructions of the patient’s bone surfaces. These reconstructions allowed determination of the most appropriate graft shape by virtually superimposing an iliac crest bone block onto the glenoid, thereby defining the optimal size and morphology of the graft. Based on this planning, the harvesting of a tricortical graft was secured through the design of a patient-specific cutting guide, whose position was defined using reliable and easily identifiable landmarks on the anterior superior iliac spine. This virtual simulation also enabled accurate preoperative measurement of screw length, ensuring bicortical fixation on the glenoid (Figure 2). Once the surgeon validated the virtual simulation, the iliac crest cutting guide was 3D printed and packaged for surgery.

Surgical technique

Graft Harvesting

**Figure 3:**
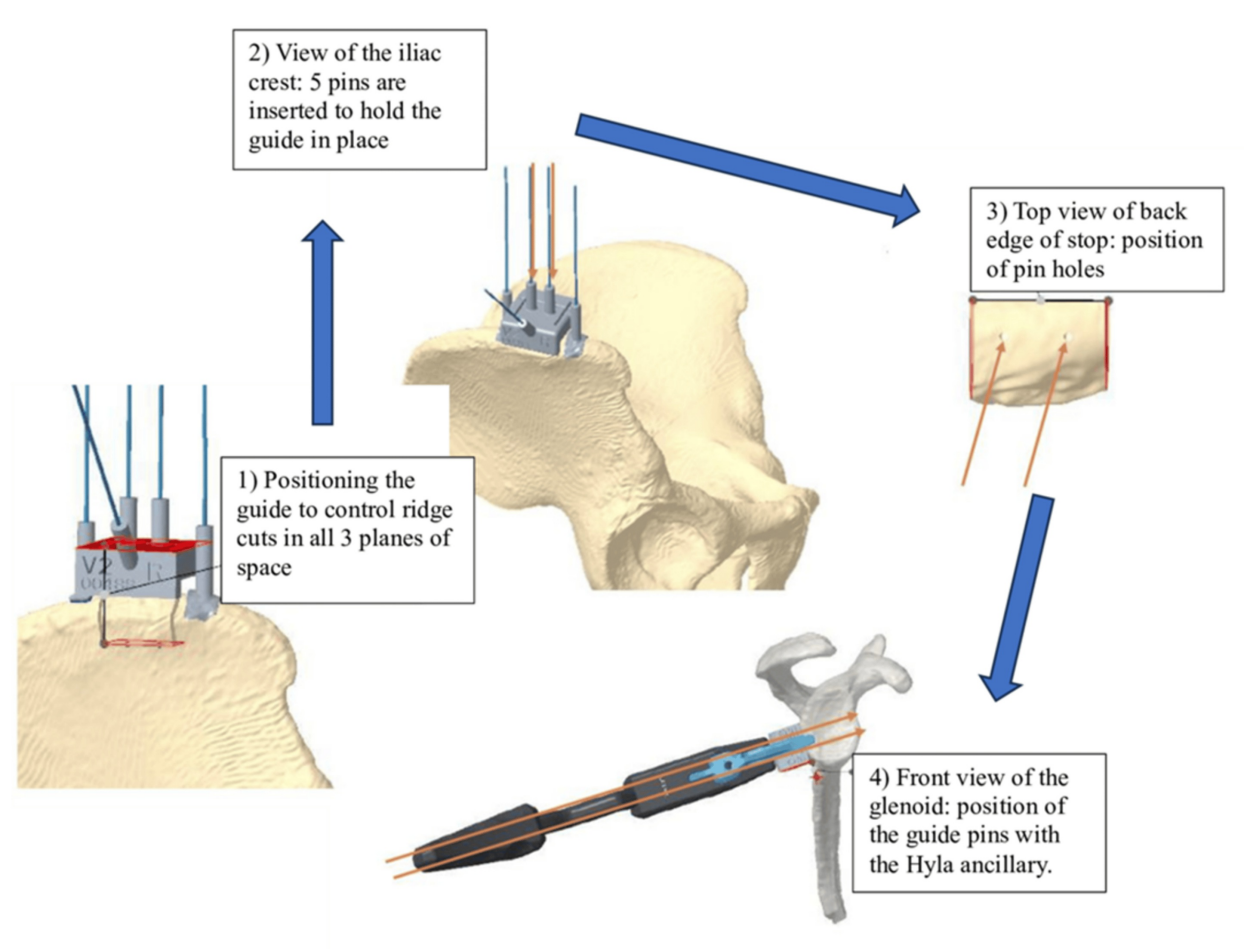
Pins previously positioned during iliac crest harvesting are inserted into the gun guide Use of the patient-specific cutting guide and pin positioning during iliac crest graft harvesting. (1) Guide positioning to control ridge cuts in all three planes of space. (2) Iliac crest view with five pins inserted to secure the guide. (3) Superior view of the posterior edge of the graft showing pin holes. (4) Anterior glenoid view demonstrating the positioning of guide pins with the HyLa ancillary device (pins indicated by red arrows).

The incision was made 2 cm behind the anterior superior iliac spine (ASIS), along the crest. The oblique and transverse abdominal muscles were removed. The patient-specific guide was placed on the upper edge of the ridge as recommended by the dedicated planning, with a distance mark from the ASIS. The guide was secured by five 1.2 mm pins and allowed the creation of 4 cuts in the tri-cortical graft. The two upper guide pins were retained to position the graft in the HyLa pistol guide (Figure 3).

Positioning the Bone Block

**Figure 4:**
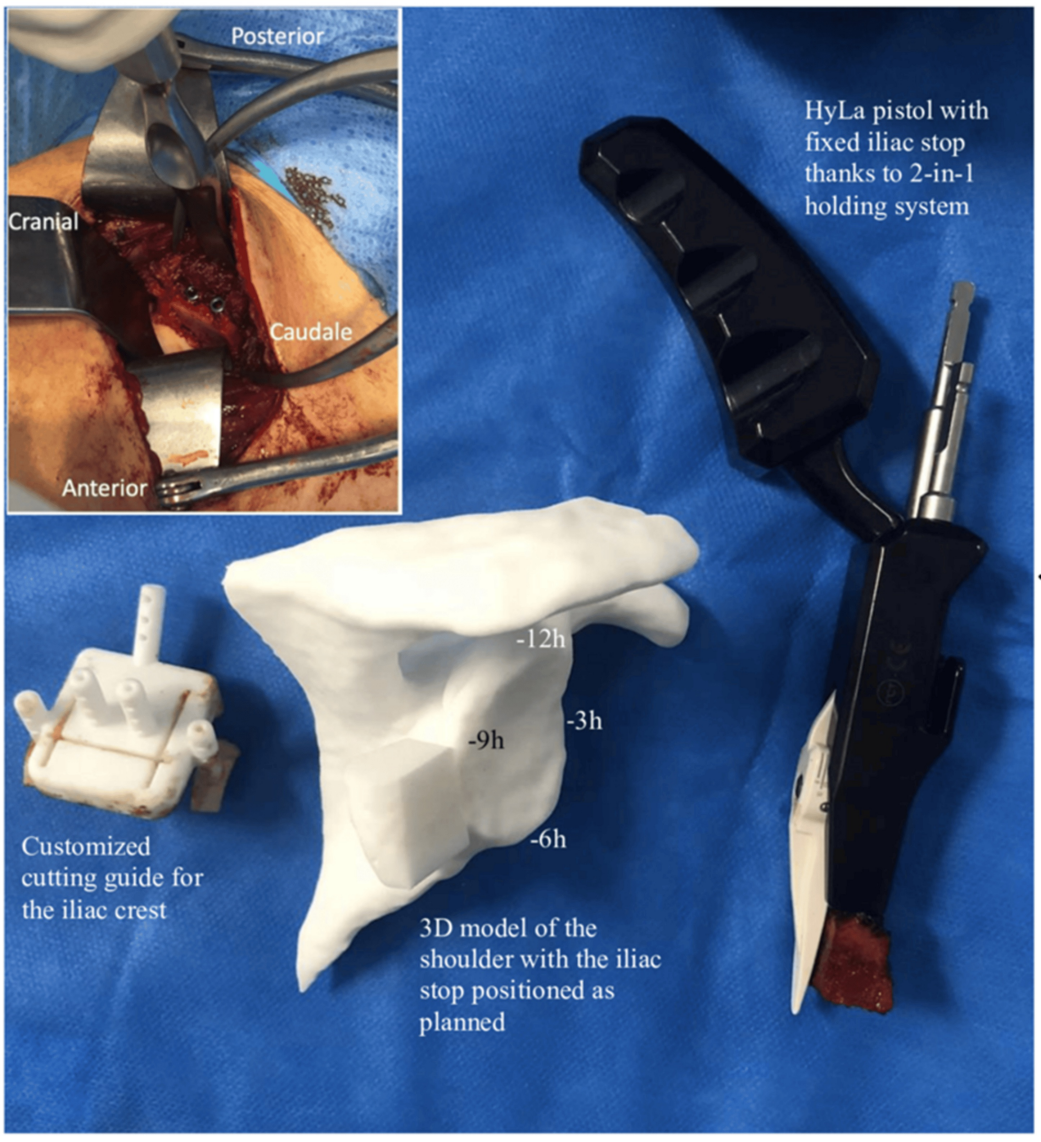
Intraoperative views with presentation of the iliac crest cutting guide Intraoperative and 3D views illustrating the posterior shoulder bone block procedure. 3D model of the shoulder with planned positioning of the bone block and HyLa gun, and view of the bone block secured by two screws at the end of the procedure. (A) 3D model of the shoulder showing planned iliac crest graft positioning with reference clockface landmarks (−12h, −3h, −6h, −9h). (B) Customized iliac crest cutting guide used for standardized graft harvesting. (C) HyLa pistol guide securing the iliac graft with the 2-in-1 holding system. (D) Final intraoperative view showing the bone block fixed to the posterior glenoid with two screws.

A posterior shoulder approach was performed with a 7-cm vertical incision, located 2 cm medial to the posterior angle of the acromion. The adipose tissue was dissected down to the deltoid fascia, and the deltoid was split along its fibrous axis to expose the infraspinatus and the lesser tuberosity. Dissection of the interval between these two muscles revealed the posterior glenohumeral capsule, which was generally of poor quality and distended. The capsule was opened parallel to the joint line, and the glenoid labrum was reinserted with a 1.8-mm anchor (FiberTak, ARTHREX, Naples) after quality assessment. A narrow Fukuda retractor was placed in the joint to push the humeral head forward, and the posterior surface of the glenoid was prepared by abrasion with an oscillating saw. The iliac bone block, previously mounted on the HyLa guide, was then positioned on the posterior surface of the glenoid between the 6 and 9 o’clock positions, with an adjustable intra-articular tab ensuring that the graft did not protrude. Once in place, the two pins were advanced into the glenoid. The unicortical preparation of the glenoid facilitated the insertion of two 3.5-mm self-tapping, self-piercing screws, whose lengths had been predetermined during 3D planning (Figure 4).

## Discussion

Three patients underwent posterior shoulder bone block surgery using the previously described technique. Postoperative CT scans were performed to confirm correct graft positioning. The rehabilitation protocol allowed immediate passive pendular movements, with additional passive mobilization introduced at three weeks and active mobilization at seven weeks. Two military patients successfully returned to work at six months, which corresponds to the usual time for consolidation, and one amateur rugby player resumed sports at eight months, returning to physical activity without discomfort (e.g., strength training exercises such as push-ups and pull-ups at the same level as before surgery). No discomfort was reported at the iliac crest donor site, and no recurrence of instability was observed. Overall, the postoperative outcomes were favorable, with no instability or subluxation recurrence. Joint congruence was preserved, as confirmed by CT analysis. Taken together, these results highlight the overall success of this procedure, from surgical execution to functional recovery. Posterior shoulder stabilization techniques were first described more than 70 years ago and have continued to evolve despite their relatively rare indications. While their use remains mainly limited to traumatic cases, posterior instability associated with glenoid bone loss and failure of rehabilitation [15,16] has created the need for customized surgical solutions. Consequently, numerous techniques have been developed to simplify and secure such procedures [17], paving the way for the Latarjet anterior shoulder stabilization. In this context, Doursounian et al. [18] reported greater reproducibility of the procedure through the use of PSI, and more recently, La Banca et al. [13] described a technique for harvesting and placing the coracoid during the Latarjet procedure using only 3D-printed instruments. For anterior shoulder bone block procedures, the accuracy of patient-specific surgery is increasingly being demonstrated [19,20]; however, no studies to date have reported on the use of 3D modeling and customized cutting guides for posterior shoulder bone block. In line with the literature, our technique is innovative, representing, to our knowledge, the first such description, and demonstrates the advantages of this technology: precision and reproducibility. PSI combined with 3D planning also makes it possible to anticipate specific challenges, such as unexpected glenoid bone loss or variations in iliac crest morphology. This approach provides a simple yet detailed framework for addressing a procedure that remains rare in current practice.

## Conclusions

This study presents an innovative tool and surgical planning approach to assist in the design, cutting, positioning, and fixation of the iliac graft in posterior bone block procedures for shoulder instability. We consider the three-dimensional design of the graft and the use of customized 3D guides as a natural evolution of conventional orthopaedic surgical planning and a significant improvement in technical execution. Despite these promising results, we acknowledge that larger clinical series will be required to better evaluate the accuracy and reproducibility of this technique. In the same line, we are currently developing a patient-specific intra-articular tab to facilitate graft placement along the glenoid edge. Another potential refinement of this approach would be to incorporate the assessment of on-track and off-track bone lesions into the planning process.
